# Supplementary material for: Extracellular Vesicles Derived from MDA-MB-231 Cells Trigger Neutrophils to a Pro-Tumor Profile
Source: Cells. 2022 Jun 9;11(12):1875. doi: 10.3390/cells11121875 (PMC9221190; doi:10.3390/cells11121875)
Supplement: Supplementary file 1 [file cells-11-01875-s001.zip › cells-1704373-supplementary.pdf]

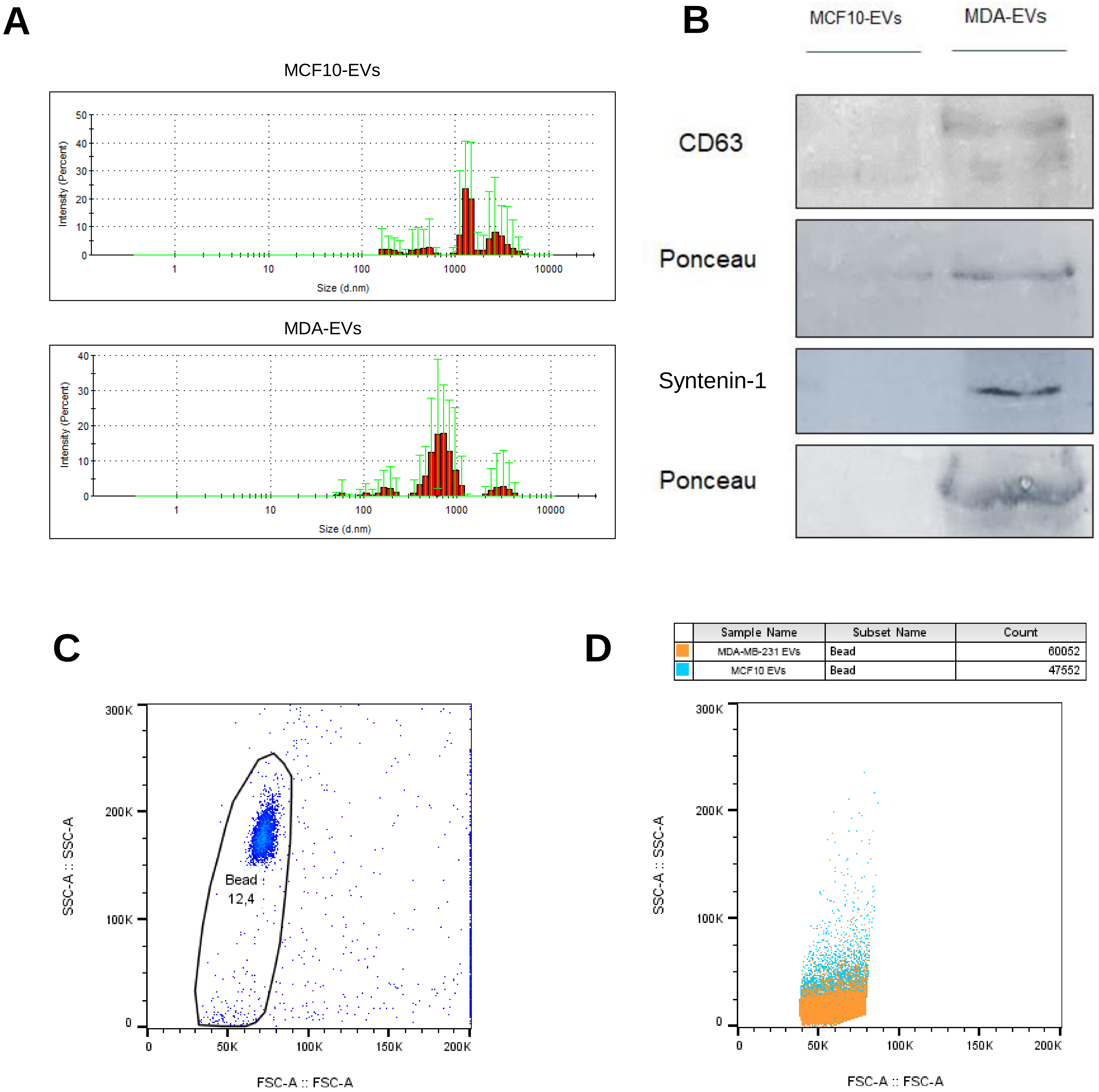

Figure S1: Characterization of extracellular vesicles (EVs) released from MFC10 and MDA-MB-231 cells. (A) Zetasizer Analysis: the size distribution in nanometer scale of extracelular vesicles was determined using the dynamic light scattering (DLS, Zetasizer Nano-S90, Malvern, Worcestershire, United Kingdom) technique at 25°C with scattering angle 90° in aqueous solution. (B) EVs were lysed using RIPA buffer and the protein extracts were separated using SDS-PAGE with 12% acrylamide gels to identify the expression of exosomal markers such as CD63 and syntenin-1. In the representatives images, contrast and brightness were adjusted to better visualize the bands. (C) Gating strategy performed comprising an area smaller than the bead of 2 µm. (D) Micropartices quantification of MCF10 and MDA-MB-231 was performed through annexin V posivite events using flow cytometry. C and D were analyzed in FlowJo 10.8.1 software.
